# Validation of At-Home Application of a Digital Cognitive Screener for Older Adults

**DOI:** 10.3389/fnagi.2022.907496

**Published:** 2022-07-01

**Authors:** Melissa Arioli, James Rini, Roger Anguera-Singla, Adam Gazzaley, Peter E. Wais

**Affiliations:** ^1^Department of Neurology, Neuroscape and Weill Institute for Neurosciences, University of California, San Francisco, San Francisco, CA, United States; ^2^Ochsner Health, New Orleans, LA, United States; ^3^Departments of Physiology and Psychiatry, University of California, San Francisco, San Francisco, CA, United States

**Keywords:** remote digital, cognitive aging, cognitive screener, neuropsychological, LTM

## Abstract

Standardized neuropsychological assessments of older adults are important for both clinical diagnosis and biobehavioral research. Over decades, in-person testing has been the basis for population normative values that rank cognitive performance by demographic status. Most recently, digital tools have enabled remote data collection for cognitive measures, which offers the significant promise to extend the basis for normative values to be more inclusive of a larger cross section of the older population. We developed a Remote Characterization Module (RCM), using a speech-to-text interface, as a novel digital tool to administer an at-home, 25-min cognitive screener that mimics eight standardized neuropsychological measures. Forty cognitively healthy participants were recruited from a longitudinal aging research cohort, and they performed the same measures of memory, attention, verbal fluency and set-shifting in both in-clinic paper-and-pencil (PAP) and at-home RCM versions. The results showed small differences, if any, for how participants performed on in-person and remote versions in five of eight tasks. Critically, robust correlations between their PAP and RCM scores across participants support the finding that remote, digital testing can provide a reliable assessment tool for rapid and remote screening of healthy older adults’ cognitive performance in several key domains. The implications for digital cognitive screeners are discussed.

## Introduction

Cognitive performance often declines as a part of the normal process of healthy aging (Jagust, 2013), as evident by measurable deficits in the critical capabilities for episodic memory, working memory, verbal fluency, and executive control in visual attention and set-shifting that do not inevitably result in dementia (Oh et al., 2012). Findings from longitudinal studies with older adults suggest that approximately 10–18% of the population, once past 70 years of age, will experience marked deficits in one or more of these critical cognitive domains sufficient to meet the clinical criteria for diagnosis of mild cognitive impairment (MCI) (Albert et al., 2011; Peterson et al., 2018; Gillis et al., 2019). Taking into account the broad spectrum of cognitive performance in aging populations, which ranges from subjective cognitive impairment to MCI to mild dementia (Petersen, 2004; Peterson et al., 2014; Salthouse, 2019), the development and validation of tests that comprise robust individual cognitive assessments are critically important for both diagnostic applications in clinical care and biobehavioral research. Today, digital technologies offer important advancements that have the potential to improve cognitive screening tools for applications in clinical treatment, therapeutic intervention, and basic behavioral research.

Neuropsychologists have developed several different batteries of cognitive assessment tools using in-person testing to assess the performance of older adults. Each of these neuropsychological batteries aims to place results for an individual older adult’s capabilities within the context of the population distribution of scores, such that assessment of the individual’s performance is normalized for age and, primarily for purposes in basic research, also for elements of demographic status. These normative scores are tracked by age-band, sex, and demographics, and they do not include allowances for developmental learning or reading disorders that can exacerbate the appearance of an older adult’s cognitive difficulties (Lebowitz et al., 2016). The California Verbal Learning Test II (CVLT II; Delis et al., 2000), The Rey Auditory Verbal Learning Test (RAVLT), and Mini-Mental State Exam (Folstein et al., 1975) are examples of broadly used neuropsychological exams that are administered by a trained clinician, in person, as paper-and-pencil (PAP) tests. An individual’s raw results from these tests are then related to normative scores organized by age-band, sex, and other demographic factors to characterize the individual’s cognitive status relative to average performance for older adults of similar age and education. In clinical application, a patient’s history of developmental learning, reading or other behavioral disorders needs to be taken into account as a factor in the interpretation of their results versus normative scores (Fletcher and Grigorenko, 2017).

Early examples of computer-based (i.e., digital) cognitive assessment tools aimed to ease the significant obstacle encountered by populations in underserved communities. Whether due to geographic or economic barriers, these populations had limited or no practical access to neurocognitive testing (Zakzanis and Azarbehi, 2013). More recent projects have focused on the advantages of automation in administering neurocognitive testing (Koenig et al., 2019; Sacco et al., 2019). These proof-of-concept studies, which collected data using in-person testing with trained technicians, have reported fair correspondence between the digital and analog test versions (Bjorngrim et al., 2019; Sacco et al., 2019; Vermeent et al., 2021), although findings vary between particular cognitive domains. Results have shown strong correlations between digital and analog raw scores in measurements of older adults’ performance on immediate verbal memory tests, but moderate to weak correlations in their performance on verbal tests of delayed recall and digit span, as well as executive control during set-shifting (Bjorngrim et al., 2019; Sacco et al., 2019; Mackin et al., 2021; Vermeent et al., 2021).

In the evolving digital era, software application programming interfaces (API) have been developed that promise broad utility for tablet computers in the administration of cognitive assessment tools. One advantage of digital cognitive assessment tools is automation in administering and scoring. However, this may be viewed as an important limitation as clinicians often depend upon nuanced observations gained during the interpersonal assessment to form a gestalt diagnostic opinion. Another advantage of digital data collection, perhaps most significantly for the advancement of behavioral research, is improved access for participants *via* the convenience of remote testing at home. Collection of data from far larger samples with broader social diversity has become a key aim given the increasing importance for population mental health research and cognitive aging in particular (Anguera et al., 2016; Peterson et al., 2018; Koenig et al., 2021). Important societal changes will be needed, however, to take broad advantage of the advent of tablet computers at more affordable price points that enable accurate speech recognition API’s. The promise of these digital tools to improve cognitive assessment techniques in the future, including (i) enlarging and diversifying the population sample for normative scores and (ii) expanding the availability of simpler, more accessible and valid characterization tests, depends upon much broader realization of open-access software, availability of free internet, and efforts to increase ease of use older adults (Martinho et al., 2022).

Tablet computers operating on iOS or Android platforms incorporate duplex audio systems that can practically support speech recognition APIs, which enable digital assessment software to mimic traditional in-person PAP tests during both the instruction and participant response phases of each task. Therefore, digital cognitive assessment applications that use narrated instructions and automated transcription of a participant’s spoken answers or responses drawn on the tablet desktop promise utility to closely match the procedures of in-person tasks such as CVLT-II, RAVLT, and similar standardized tests (Koenig et al., 2019; Sacco et al., 2019).

As demonstrated by the stability of speech recognition API’s to serve in popular digital assistants such as Siri, Alexa, and Google Home, as well as many millions of customer service contacts *via* telephone each day for airlines, retail conglomerates, and local utilities, this functionality is available and reliable for adoption in a digital cognitive assessment application (i.e., Remote Characterization Module, or RCM) that utilizes broadband internet connections to link with a participant testing with a tablet application at home. Their data uploaded to a HIPAA-compliant secure data server can be transcribed, scored, and archived for analysis. Tablet computers are the desirable platform for digital cognitive assessment applications because standardization of the necessary audio and recording functions can be hardcoded into the RCM that the participant downloads for home use and the large viewable screen better facilitates graphical tasks. Speech recognition APIs are all very sensitive to input signal standardization, which precludes web-browser-based plugins that may operate across many different combinations of computer hardware and operating systems.

Our motivation was to use the most modern digital tools in the development of RCM to achieve broad utility for basic behavioral research in terms of pre-screening and stratification of older adults by their cognitive performance. To assess the critical higher cognitive capabilities engaged during most behavioral research experimental procedures, we selected tasks measuring verbal working and long-term memory, verbal fluency, and cognitive control in set-shifting/planning. Our UCSF Neuroscape Digital Media Studio engineers developed RCM on a Unity platform and first deployed the program on an iOS device using an Apple speech recognition API. Nine tasks were programmed to mimic the instructions and test procedures from standardized measures in CVLT II and Trail Making Test Part B (Table 1A) such that the total time-on-task for an older adult running RCM would not exceed 25 min, after spending 5–10 min on iPad set-up, orientation, and practice for the operation of the speech recording steps necessary to capture their data in real-time.

**TABLE 1:** Lists of measures participants completed and normative performance.

Characterization of participants		
*n* = 40 (23 female)	76.7 ± 6.7 years old	17.3 ± 1.3 years education
**(A) Tests in bedside neuropsychological screen (NP)**		
Montreal mental status exam		Mathematical calculations
CVLT-II verbal immediate memory		Sentence repetition
CVLT-II verbal recall after 30 s delay		Verbal agility
CVLT-II verbal recall after 15 min delay		Sentence comprehension
CVLT-II cued recall		Lexical fluency
CVLT-II verbal recognition memory		Semantic fluency
Modified trails (numbers and days of the week)		Abstraction
Drawing design fluency		Boston naming test
Visuospatial ability: Benson figure		Stroop color naming
Verbal digit span forward		Geriatric depression scale
Verbal digit span backward		
**(B) Mean normative scores in tests selected for RCM**		
CVLT II		
Total immediate recall (80 words)		*z* = 0.8 ± 1.0
Short-delay free recall (16 words)		*z* = 1.2 ± 1.1
Long-delay free recall (16 words)		*z* = 1.0 ± 1.1
Lexical fluency (B words, 60 s)		*z* = 0.4 ± 0.8
Semantic fluency (vegetables, 60 s)		*z* = 0.5 ± 0.8
Modified trails B (7 days, 8 numbers)		*z* = 0.2 ± 0.9
Verbal digit span		
Forward		*z* = 0.2 ± 1.0
Backward		*z* = 0.3 ± 1.2

***(A)** All participants completed a neuropsychological test battery (i.e., NP) in the UCSF Memory and Aging Clinic as healthy controls in the Hillblom Longitudinal Aging Network cohort. **(B)** The measures from the NP that were selected for development into digital presentation in RCM are listed along with the mean age-normalized scores (±SD) from participants’ NP.*

We planned to validate RCM as a reliable cognitive assessment tool by comparing participants’ scores from in-person paper-and-pencil tests (i.e., PAP) collected in a formal laboratory setting to their scores on the analogous digital task collected remotely from the laboratory. Our design and analysis were concerned with testing the similarity in participants’ performance between PAP and RCM versions of each task, and also the comparability of individual differences in participants’ scores. Consistent with several recent and relevant reports that have compared digital methods of cognitive screening (Bjorngrim et al., 2019; Domen et al., 2019; Koenig et al., 2019; Sacco et al., 2019; Mackin et al., 2021; Vermeent et al., 2021), we use the similarity of mean PAP and RCM scores on a task to inform construct validity for the RCM version of that measure, and the comparability of participants’ individual differences (PAP versus RCM) to inform inter-method reliability and validity of the RCM version of that measure. Our hypothesis was that pairwise comparisons of the data collected on each task (i.e., digital versus PAP versions) would not show statistically meaningful differences between versions, and we would interpret this finding as confirmation that participants experience the remotely collected RCM and in-person PAP tasks as being highly similar. We also hypothesized that an analysis of individual differences in scores on each task would show robust positive correlations between the RCM and PAP data, which we would interpret as key evidence validating RCM both as a digital version for testing constructs of the PAP tests and as an effective remote cognitive assessment tool.

## Materials and Methods

### Participants

Forty older adults (mean age 74.5 ± 6.5 years, 23 females) with average cognitive capabilities for their age participated in this study. Their mean education was 17.3 ± 1.3 years. The participants were recruited from the UCSF Hillblom Longitudinal Aging Network cohort of healthy controls,^1^ which was organized to conduct serial annual neurocognitive evaluations to confirm no or only minor memory problems without evidence of a neurodegenerative disease or other major health condition. Inclusion criteria were fluent speakers of English, normal or corrected-to-normal vision, and residency within a 50-mile driving distance from our campus. All of the enrolled participants had completed their most recent neuropsychological assessments at the UCSF Memory and Aging Center within the past 2 years, before on-site examinations were suspended in accordance with public health restrictions during the COVID-19 pandemic. Participants gave their informed consent in accordance with the Declaration of Helsinki and the Institutional Review Board of the University of California, San Francisco, and were then provided an iPad tablet computer with the RCM application loaded. The tablet was delivered and picked up from their residence. All methods were carried out in accordance with relevant guidelines and regulations for experimental protocols approved under UCSF IRB #20-31812. Following their RCM digital assessment procedure, 20 of the participants also completed virtual tele-neurology examinations, and those data are not part of the study and not included here.

### Neuropsychological Testing

All participants enrolled in the study, prior to their recruitment, had already been administered the standardized UCSF Memory and Aging Center Bedside Neuropsychological Screen (Table 1A), which assessed executive and memory functions, and depression. Each participant scored >−1.0 SD of normative values for their age on each of those tests, had a Clinical Dementia Rating (CDR) score of 0 and MMSE score greater than 25, reported no cognitive decline during the previous year and was evaluated as showing no evidence of neurodegeneration by a panel of experts at the UCSF Memory and Aging Center. Mean normative scores from the participants’ bedside screens, accounting for sex and education, are reported in Table 1B for each of the eight tasks selected for inclusion in the RCM procedure.

### Overview of Experiment Procedures

The eight active RCM tasks included measures that assessed LTM, WM, verbal fluency, and set-shifting with direction of attention.

The CVLT II measured LTM in three word-recall tasks after immediate, 2- and 20-min retention intervals (Delis et al., 2000). The Verbal Digit Span Test measured WM in two tasks, forward and backward repetitions of strings of single digits, according to the procedures of the Wechsler Adult Intelligence Scales (Wechsler, 1981). Verbal fluency measures were tested in two standardized domains, using the Semantic Fluency test to assess production of category-specific nouns and using the Lexical Fluency test to assess production of words while switching semantic categories (Eslinger et al., 1985; Troyer et al., 1997). The Modified Trail Making Task-B measured control of attention and executive functions in a set-shifting using a modified 15-step procedure (Reitan, 1958).

Recruited participants completed an eligibility questionnaire *via* telephone, which included an assessment by a behavioral neurologist that they did not have notable changes in cognitive or medical history since their last formal evaluation as part of the Hillblom Longitudinal Aging Network. The questionnaire also confirmed they had internet access and a Wi-Fi-enabled smartphone or computer with a camera and microphone to support a Zoom video call. All consent materials were signed *via* DocuSign. Before their study appointment, a member of the study team dropped off the study materials at the participants’ residence. The study materials contained a secured iPad (9.7 inch fifth generation) with the RCM application downloaded, an iPad charger, and instructions for how to unlock and connect the iPad to their home Wi-Fi.

The study session was completed in the participant’s residence using Zoom video conferencing over an internet connection, remotely from our laboratory and experimenters. The session lasted approximately 45 min and involved a review of setting up the iPad onto the participant’s Wi-Fi network and an overview of the RCM application. Participants were told that they would complete nine tasks and that the programmed RCM narrator would provide all task instructions. Once the set-up orientation was complete, the researcher muted themselves and remained on the Zoom call to observe the session, take notes, and assist in the event of any technical problems. The experimenter observed the participant interact with RCM and made sure that the participant did not make written notes during the tasks.

### Narration of Instructions and Remote Characterization Module Tutorial

**FIGURE 1:**
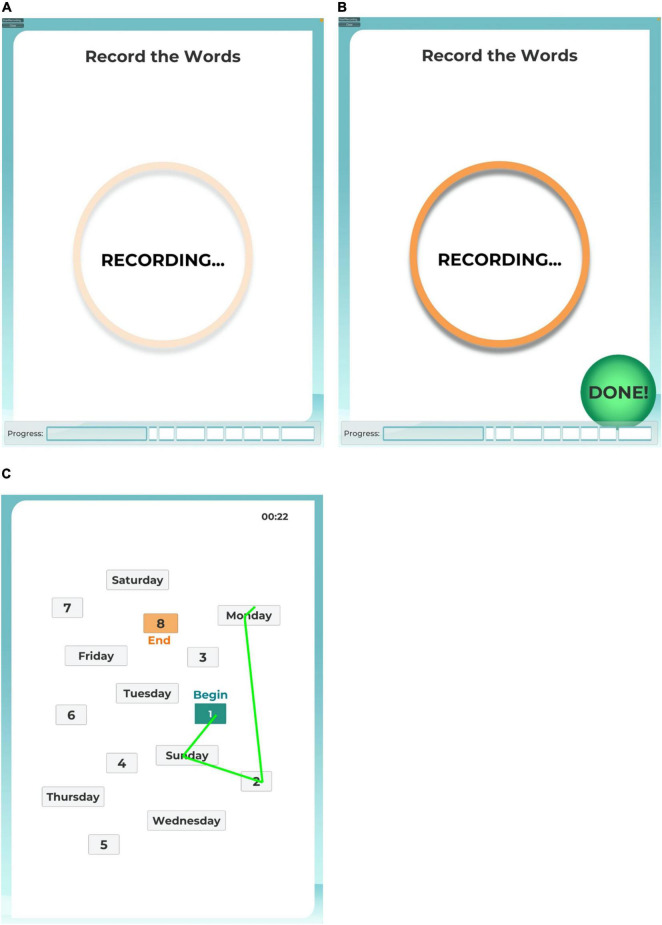
iPad screen images. The user interface provided participants with an animated recording reminder during the active task sessions **(A)** and a control button to end a task session before the time limit **(B)**. For task 8, the user interface provided participants with a map of numbers and days of the week to connect by drawing a line with their finger tip **(C)**.

Task instructions were narrated by a digitized female voice speaking at a slow, conversational pace. For each task, the procedure was explained, and then a practice tutorial followed. The application was programmed to score the practice result in real time, for which the result was then related to the participant. A correct result was necessary to move forward to the actual task, or the narration said “nice try, let’s do it again as,” before repeating the instructions again. After each set of instructions and tutorial, the participant was cued to start the task by touching a button icon at the bottom of the iPad screen indicating they were ready, and then the iPad screen showed a pulsing “RECORDING” circle during the active task period (Figure 1A). The participant ended the task by pressing a “DONE” button icon at the bottom of the iPad screen (Figure 1B), or the task timed out according to the response duration limit from the respective analog test. If a task timed out, the RCM program automatically advanced to the next task.

For the six tasks requiring the participant to speak a list of words (i.e., immediate word recall, filler trial between immediate and short-delay word recall, short-delay and long-delay word recall, lexical fluency, and semantic fluency), participants were instructed to say “comma” in between each word spoken in their answers. The word “comma” was programmed for the six word-based tasks to cue the automated scoring algorithm to compare the preceding spoken word as an individual answer to the allowable trial responses for that task. In this fashion, the scoring algorithm would show that the participant was reciting a list of words, not a continuous sentence, and would not transcribe the word “comma.” In pilot experiments with the Apple speech recognition API, this allowance produced a reliable recording and automated scoring of words as a list, and it avoided introducing a button press or other spacing step between words that would have added a modality not involved in the analog PAP tests. Before the first task, a tutorial led the participant to practice saying “comma” between words on a shortlist. Participants repeated this practice until they made no errors. Once the practice was complete, RCM moved on to Task 1.

### Task Procedures

The order of the tasks in RCM was matched to the order of the tasks that participants had completed during their PAP sessions, which followed the standardized sequence of the UCSF Memory and Aging Center Bedside Neuropsychological Screen used for the Hillblom Longitudinal Aging Network. Because of the delay intervals required between the CVLT II Immediate Word Recall sequences and the Short-Delay, and then Long-Delay Free Recall tasks, the WM, verbal fluency and Trails Making-B tasks were ordered as described below.

Task 1 in RCM mimicked CVLT II Immediate Word Recall, which includes five successive sequences of learning a list of 16 common nouns and then immediately repeating them back as a list from memory. After hearing the narration of the 16-item word list on each learning trial, the participant was instructed to recall and speak as many words from the list as they could. The response period timed out at 60 s, or sooner if the participant pressed the DONE button.

Automated scoring of each of the five sequences compared recorded responses against the 16 target words. Responses other than the first mention of each of the 16 possible hits were recorded as intrusions or repetitions in each sequence. For Task 1, the possible total score was 80 hits.

Following the Immediate Word Recall task, Task 2 instructed the participant to spell the word “WORLD” forward and then backward. This procedure served as a brief filler task for the short-delay recall task that followed.

Task 3 mimicked CVLT II Short-Delay Free Recall, which instructs the participant to freely recall and speak all of the words they remember from the list of 16 common nouns learned in Task 1. The response period timed out at 60 s, or sooner if the participant pressed the DONE button. Responses other than the first mention of each of the 16 possible hits were recorded as intrusions or repetitions, and the possible score in Task 3 was 16 hits.

Task 4 mimicked the Verbal Digit Span forward test in which participants hear a random sequence of single-digit numbers (i.e., numbers 1 to 9, inclusive) and then immediately repeat back the numbers in the same order. The Digit Span procedures were adapted from the UCSF Memory and Aging Center Bedside Neuropsychological Screen. Participants were not instructed to say “comma” between numbers because the tests targets were defined in the automated scoring algorithm as words for the sequence of numbers, including homonyms for “2, 4, and 8.” The algorithm did not score or transcribe any spoken words other than numbers 1 through 9. In this fashion, the verbal working memory span probed by the task was not burdened by keeping in mind or producing any information other than the numbers spoken by the narration.

Beginning from a span of three digits, Task 4 advanced the span by one digit when a trial was repeated correctly. If repeated incorrectly, the application administered a second trial of the same span length. The task was discontinued when two successive trials of a span length were answered incorrectly. A participant’s score for Task 4 forward was the longest span repeated correctly before two successive incorrect trials, up to a maximum span of nine digits.

Task 5 mimicked the Verbal Digit Span backward test in which participants hear a random sequence of single-digit numbers and then immediately repeat back the numbers in the reverse of order spoken by the narration. In all other respects, Task 5 procedures and scoring were the same as in Task 4.

Task 6 mimicked the Lexical Fluency test, which instructs participants to say as many words as possible that begin with the letter B, other than proper nouns, in 60 s. The task procedures were adapted from the UCSF Memory and Aging Center Bedside Neuropsychological Screen. Participants were instructed that answers modifying the same root word, for example, with a modified ending to change verb tense or increase to a plural form, would not count as a hit but instead a rule violation. Participants were instructed to say “comma” between each word. The response period timed out at 60 s, or sooner if the participant pressed the DONE button. Responses were scored as hits, violations, or repetitions.

Task 7 mimicked the Semantic Fluency test, which instructs participants to say as many words as possible that name an animal in 60 s. The task procedures were adapted from the UCSF Memory and Aging Center Bedside Neuropsychological Screen. Participants were instructed that answers modifying the same root word to plural form would not count as a hit but instead a rule violation. Participants were instructed to say “comma” between each word. The response period timed out at 60 s, or sooner if the participant pressed the DONE button. Responses were scored as hits, violations, or repetitions.

Task 8 mimicked the Trails Making Test B, as adapted from the Modified Trails (Numbers and Days of the Week) procedure used in the UCSF Memory and Aging Center Bedside Neuropsychological Screen. For this task, participants were instructed to use their finger on the iPad’s touch screen to draw a line to connect alternating numbers to days of the week, in order beginning with 1 and Sunday. Before beginning the task, participants completed a tutorial exhibiting correct and incorrect responses in connecting the numbers and days. Connecting points in the correct sequence changed the drawn line to green (Figure 1C). If an error was drawn, the incorrect line flashed red, and the participant was instructed by the narration to try again, starting from the previous correct point. The response period timed out at 120 s, or sooner if the participant pressed the DONE button. The score recorded for this task was the elapsed time to connect all 15 points in the correct sequence.

Task 9 mimicked CVLT II Long-Delay Free Recall, which instructs the participant to freely recall and speak all of the words they remember from the list of 16 common nouns learned during Task 1. In our RCM procedure, the delay interval between the fifth repetition in Task 1 and the recall test in Task 9 was approximately 10–12 min.

Participants were instructed to say “comma” between each word. The response period timed out at 60 s, or sooner if the participant pressed the DONE button. Responses were scored as hits, violations, or repetitions, and the possible score in Task 9 was 16 hits.

### Analysis

Data recorded by the RCM application were reviewed by the experimenter, who witnessed the participant’s remote session *via* Zoom in order to compare the automated score on each task with notes taken in real-time during the session. Per this review, six participants had caught their failures to press the “start” button and enable recording in Tasks 3 or 8. In each of these six cases, the participant paused making responses once recognizing that the “RECORDING” symbol was not showing (Figure 1A), and then pressed the visible “START” button to restart their task response that was recorded in the data file. In post-session debriefing with the experimenter, each of these six participants mentioned their errors and how they were resolved.

The goal of comparing experimenter notes to the automated scoring file was to control any quality problems in the data arising from a faulty internet connection during a task. In the analysis, we verified that each participant’s data file for Tasks 1, 2, 3, 6, 7, and 9 showed only one word as a hit or as an intrusion per counted value, which effectively verified that the API was correctly parsing the participant’s replies into individual words. Any corrected responses spoken by a participant after a false start during an RCM task were not credited as correct answers, which is a procedure that is sometimes subjectively applied during in-person neuropsychological assessments in the clinic. Data were analyzed and statistics calculated using IBM SPSS Statistics release 20.0, and the threshold of statistical significance for false positives was set at *p* < 0.05.

As a result of the extended interval between the participants’ previous in-person assessments at the UCSF Memory and Aging Center and their RCM session (mean interval = 23.87 ± 3.8 months), nine participants had advanced to the next higher age-band of normative scoring for the standardized PAP tests. Two participants had advanced to the 70–79 years age band, and seven participants had advanced to the 80–89 years age band.

### Data Availability

De-identified raw data from PAP measures were generated at the UCSF Memory and Aging Center, and the relevant derived data supporting the findings of this study are available from the corresponding author, upon reasonable request. The de-identified RCM data that support the findings of this study are available from the corresponding author, upon reasonable request. The RCM application developed by our research team is available to download onto a compatible iPad tablet computer, upon request to the corresponding author.

## Results

The analysis examined two key questions in the results for each task, PAP versus RCM, by using two different statistical tests. First, task similarity tested PAP versus RCM raw scores to compare the participants’ performance and establish construct validity for the RCM version of each measure, relative to the original in-clinic PAP assessment. Second, task comparability examined participants’ individual differences in a correlation analysis in order to evaluate the inter-method reliability of the RCM version to provide scores that were functionally analogous to PAP.

### Task Performance Similarity *via* Pairwise *t*-Test Comparisons

A direct comparison is presented in Table 2A for each task, including summary statistics, contrasting raw scores from the remotely collected RCM and the in-person PAP versions. Pairwise *t*-tests in each task showed a range in the results, including no differences in performance on four tasks, but significantly reduced performance on four of the RCM tasks relative to their PAP analogs. Short-Delay Free Recall, Long-Delay Free Recall, Lexical Fluency, and Modified Trail Making B tests each showed small numerical differences in raw scores between PAP and RCM, which resulted in small effect sizes (i.e., all Cohen’s *d* < 0.26). In each of those four comparisons, the PAP mean indicated numerically better performance than the RCM mean. This pattern suggests that participants may have experienced some sort of systematic difference completing the digital task at home relative to their previous in-person, PAP testing.

**TABLE 2:** Results for comparisons of PAP and RCM versions of each task.

Comparisons of raw scores by task	(A)			(B)		
Task	PAP	RCM	Pairwise	Cohen’s *d*	Pearson’s correlation	

	**Mean raw scores (SD)**					
**CVLT II**						
Total immediate recall	50.6 ± 11.3	46.3 ± 13.4	*t* = 2.26; *p* = 0.029	*0.35*	*r* = 0.54	*p* = 0.0003
Short-delay free recall	11.5 ± 3.4	10.9 ± 3.4	*t* = 1.27; *p* = 0.21	*0.17*	*r* = 0.61	*p* = 0.00003
Long-delay free recall	11.8 ± 3.5	10.4 ± 3.9	*t* = 0.05; *p* = 0.96	…	*r* = 0.58	*p* = 0.00009
Lexical fluency (B words, 60 s)	17.1 ± 3.9	17.1 ± 4.9	*t* = 0.03; *p* = 0.97	…	*r* = 0.45	*p* = 0.003
Semantic fluency (animals, 60 s)	23.4 ± 4.4	17.6 ± 5.3	*t* = 7.14; *p* < 0.001	*1.28*	*r* = 0.45	*p* = 0.003
Modified trails B (7 days/8 numbers)	31.4 ± 16.8 s	36.4 ± 27.8 s	*t* = 2.07; *p* = 0.045	*0.26*	*r* = 0.58	*p* = 0.00009
**Verbal digit span**						
Forward	7.2 ± 1.3	5.6 ± 1.4	*t* = 5.98; *p* < 0.001	*1.19*	*r* = 0.21	*p* = 0.19
Backward	5.6 ± 1.6	4.8 ± 1.4	*t* = 2.69; *p* = 0.011	*0.53*	*r* = 0.20	*p* = 0.22

*PAP is paper and pencil, RCM is Remote Cognitive Module, and pairwise t-test results are shown with effect size.Mean raw scores (±SD) are presented for each of the eight cognitive tasks for both the PAP and RCM sessions, including pairwise t-test comparisons with resulting effects of Task Similarity **(A)**. Pearson’s correlation tests compare individual differences in the pairings of task scores and indicate the Task Comparability **(B)** of each measure between its PAP and RCM versions (i.e., r-value).*

In the Modified Trail Making B set-shifting test (Task 8), four participants reported struggling to smoothly trace their index finger tips on the iPad desktop in order to quickly and correctly connect the sequence of numbers and days, and each of their scores exceeded 80 s. When their data is removed from the pairwise test, the result shows no difference between RCM and PAP mean scores (RCM mean = 32.4 ± 19.5 s; PAP mean = 30.9 ± 16.4 s; *t*_35_ = 0.74, *p* = 0.46).

Total Immediate Recall, Semantic Fluency, and Verbal Digit Span forward and backward all showed significant differences between the PAP and RCM versions of the tasks, such that mean performance was better on the former. For total immediate recall, the effect size was modest with Cohen’s *d* = 0.35. A *post hoc* analysis of the five repetitions in the immediate verbal recall task showed that pairwise differences in performance reached significance only during the fifth repetition (i.e., first through fourth repetitions, all *p*’s > 0.11; fifth repetition, PAP mean = 12.9 words versus RCM mean = 11.2 words, *t*_39_ = 2.94, *p* = 0.006).

For the Semantic Fluency and both Verbal Digit Span tasks, participants’ performance on the PAP tests was robustly superior to their RCM tests, and the effect size for each of the differences was large. These three pairwise results show which RCM tests the participants, on average, did not experience as being highly similar to the in-person PAP tests.

### Task Performance Comparability *via* Correlation Analysis

Pearson’s correlation coefficients were calculated for each task to test the comparability across participants between performance on the RCM and the PAP tests (Table 2B). The results showed strong, positive correlations between RCM and PAP on six tasks. Performance on both verbal digit span tasks, however, was weakly correlated between RCM and PAP.

**FIGURE 2:**
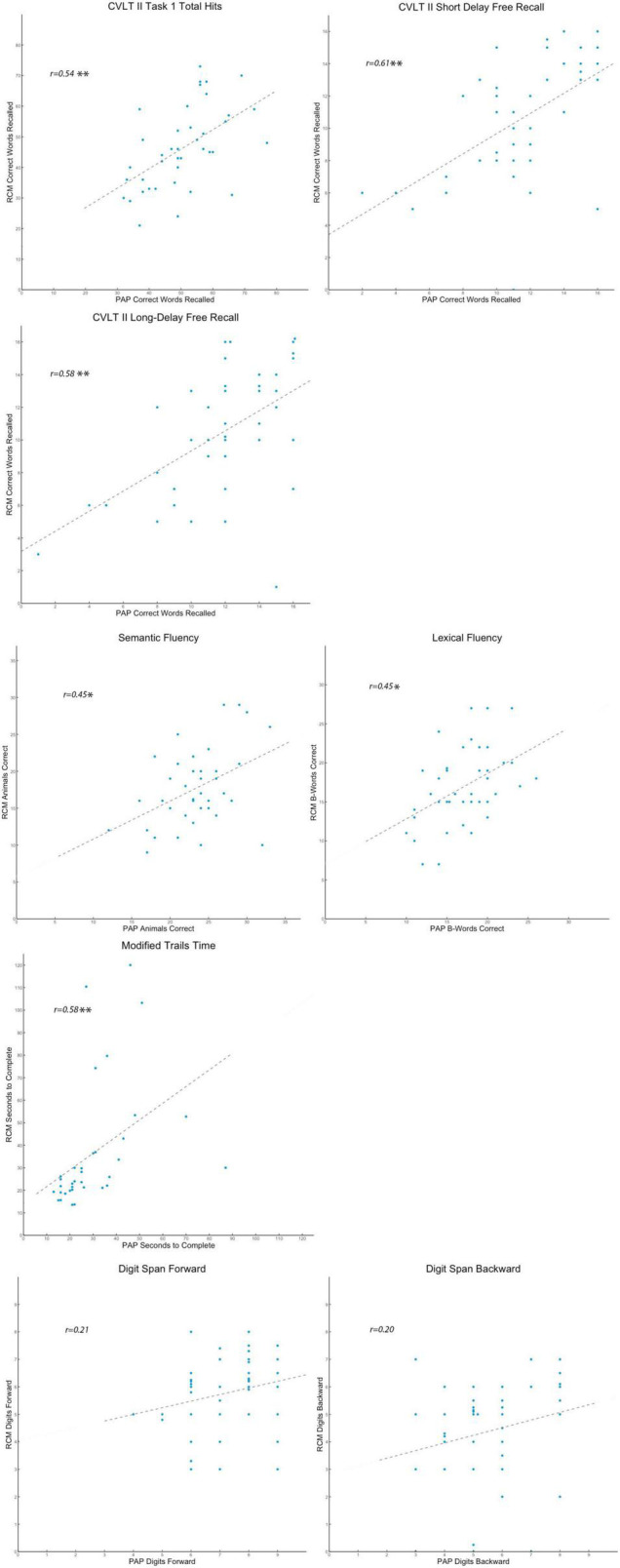
Scatter plots comparing individual differences. Presented separately for the eight standardized tasks, each participant’s raw scores are shown in scatter plots that reflect the pattern of individual differences on that cognitive measure. On each plot, participants’ RCM raw scores for the test variable are indexed by the vertical axis, while their PAP raw scores are indexed by the horizontal axis. A dashed trend line on each plot indicates the best estimate of the Pearson’s correlation (*r-*value). For the *r-*value estimates, *p < 0.05, **p < 0.01, and ***p < 0.001.

Positive correlation coefficients between RCM and PAP for short-delay free recall, long-delay free recall, lexical fluency, and modified trail making B were each consistent with the pairwise comparisons of those data, and the results provided robust confirmation that participants experienced these four RCM tasks in a manner comparable to PAP. Importantly, the correlation coefficients between RCM and PAP for Total Immediate Recall and Semantic Fluency were also positive and robust (i.e., *r* = 0.54 and *r* = 0.58, respectively, see Figure 2). The results indicate that notwithstanding pairwise differences, performance on these two tasks was reliably comparable between RCM and PAP across participants. The contrast in findings between task similarity and comparability suggests that RCM tasks for Total Immediate Recall and Semantic Fluency are valid relative to PAP, but their procedures can be adjusted to better match how participants experience those PAP tests.

The results for both verbal digit span tasks, however, suggest that RCM and PAP are somehow not testing the same construct of working memory span. Raw score differences and lack of correlation in respective results between RCM and PAP show that the former may not be a valid analog for in-person testing of verbal working memory and that further work is needed to build and validate a remote assessment tool for this critically important higher cognitive faculty.

## Discussion

Cognitive performance varies across older adults, more than for younger or middle-aged adults, because of a constellation of aging-related changes in the brain (Salthouse, 1994; Davis et al., 2008; Grady, 2012; Oh et al., 2012; Jagust, 2013; Bjorngrim et al., 2019). The effects of so-called normal cognitive aging, therefore, need to be taken into account for both the inclusion criteria for research study enrollment and the stratification of individual data in the group analysis of behavioral results. Most recently, digital tools have brought forth new alternatives for remote data collection, which present new opportunities and challenges for administering cognitive assessments and behavioral research studies on aging. Accordingly, we implemented a novel approach with a digital speech recognition API in a remote characterization module (RCM) that can rapidly assess an older participant’s memory, language fluency, and set-shifting capabilities. We discuss the validity and comparability of initial results from RCM relative to standardized PAP tests and how these findings can inform study enrollment and subsequent data analysis in future behavioral studies in aging.

Our study compared longitudinal results for older adults enrolled from a well-characterized cohort of healthy controls in the UCSF Hillblom Aging Network. They completed standardized tests of verbal memory, language fluency, working memory span, and set-shifting, both in the UCSF Memory and Aging behavioral neurology clinic using traditional PAP procedures and in a remote setting with RCM digital analogs of the same cognitive measures. The results showed that performance in three of these four cognitive domains was comparable between a participant’s scores collected with digital analogs in a remote setting and their scores on the same measures assessed in the clinic. Our findings support the application of RCM as an effective and reliable assessment tool for rapid and remote screening of healthy older adults’ cognitive performance. Importantly, our findings of comparability support the conclusion that scores from a participant’s RCM measures can be fairly compared to population-based age-normalized scores that have been established over a generation of neuropsychological testing.

The longitudinal results from RCM show that a participant’s scores on some of the digital tests are numerically lower, on average, than from their scores on comparable PAP measures administered in-person with an experimenter (Table 2A). The pairwise results in the assessments of verbal memory, language fluency, and set-shifting show systematic differences in a pattern that suggests remote testing on a tablet computer is a more challenging experience for older adults than performing the same tests in a PAP format (Hauk et al., 2018; Rabipour and Davidson, 2020). The marginal difference in difficulty between the procedures may be on account of distraction at the remote location during testing, comfort, and ease of acceptance of digital interfaces among older adults, or a combination of both possibilities (Hauk et al., 2018; Martinho et al., 2020; Rabipour and Davidson, 2020; Carlier et al., 2021).

Acceptance of digital interfaces in healthcare services among older adults is an emerging research focus (Martinho et al., 2020). In a comparison relevant to cognitive screeners, a recent study found that gamification of instructions, feedback, and rewards in the user interface led to increased interest and engagement of the participant in repetitive survey tasks (Carlier et al., 2021). Whereas our motivation here was to validate RCM using task procedures matched very closely to those that participants had followed in their PAP tests, a future RCM version incorporating personalized support and feedback to the participant based on their interaction with each cognitive task could effectively bridge the uneasiness some older adults experience with automated digital applications (Martinho et al., 2022).

Differences in the testing environments notwithstanding, however, the results did show that remote testing with RCM gives scores that are not statistically different than scores on PAP tests for delayed verbal memory, lexical fluency, and, as proposed below, set-shifting between days and numbers. Pairwise comparisons revealed no differences between participants’ RCM and PAP scores on Short-Delay Free Recall, Long-Delay Free Recall, and Lexical Fluency assessments. A small difference in pairwise scores on the Modified Trail Making B set-shifting task (i.e., Numbers and Days of the Week, Cohen’s *d* = 0.26) may reflect the more stringent RCM procedure, wherein poor performers are not assisted by the prompts of an administrator to rapidly reset their sequence to their preceding correct connection. When data from the four participants (out of the sample of 40 older adults) who struggled in tracing their Task 8 path on the iPad desktop are excluded, the trimmed pairwise comparison shows no difference in Task 8 scores between RCM and PAP.

For the five rounds in Immediate Recall (Task 1), participants’ memory for the sixteen target words increased significantly during the last round in PAP, relative to the last round in RCM, which resulted in a greater mean Total Immediate Recall score for PAP and drove a small pairwise difference relative to RCM (Cohen’s *d* = 0.35). Mean immediate recall of target words in the fifth round of Task 1 was also greater in RCM, relative to its four preceding rounds, but the increase was not nearly so robust as participants’ performance in their PAP tests. We do not have a quantifiable measure that might explain why the participants almost uniformly remembered more words in the fifth round of Task 1 in PAP, relative to their fifth round in RCM, although a contributing factor might have been the faster pace between rounds in the digitally programmed timing of RCM (Hauk et al., 2018). In-person administrators typically apply brief rest breaks nearing the end of this highly repetitive verbal memory task, whereas RCM presents a steady pace of instructions between rounds in Task 1 that may increase task demands on participants.

Scores for the two language fluency tasks produced a surprising pattern, such that lexical fluency results were equivalent while semantic fluency results were not. The cohort was screened prior to enrollment to exclude participants who reported developmental learning or reading disorders, which might have otherwise complicated interpretation of these results (Lebowitz et al., 2016). Specifically, the pairwise comparison for production of B words in Task 6 showed no difference between RCM and PAP scores, while there was a large effect for participants to produce more animal words in PAP than in RCM during Task 7. Scores in each task showed a tight correlation in individual differences between RCM and PAP. However, this supports our conclusion that the two assessment procedures should be comparable with confidence. Notably, although our results suggest that the standardized semantic task in its digital analog form was somehow more demanding on participants’ fluency (Rabipour and Davidson, 2020), the upshot for RCM Task 7 equated to mean word production within −0.50 SD of the age-normalized level for our cohort, as calculated from standard scores in the bedside neuropsychological screen.

Verbal Digit Span Tasks 4 and 5 were neither equivalent nor comparable to the PAP measures they mimicked. More importantly, the lack of correlations in individual differences between RCM and PAP on both measures show that the participants experienced the challenge to demonstrate their working memory span very differently between the remote digital task and the in-clinic task with a live administrator. By contrast, a relevant study that compared digit span assessments using an administrator in-clinic found only small effects of older adults’ higher performance on PAP than on digital tests, and more significantly, fair to good comparability between participants’ raw scores in the two tests (Vermeent et al., 2021). Interestingly, our pilot data from a new cohort of participants in a follow-up study, who ran both the RCM and PAP verbal digit span tasks in the laboratory during a one-session experiment using counter-balanced order for the method, support the interpretation that a majority of older adults are more comfortable responding to an in-person than remote assessment (Germine et al., 2019).

Assessments of the study cohort and the timing of our data collection for the RCM sessions both bare consideration in the interpretation of these results. Over the course of the study, nine of 40 participants advanced from one age-band of normative scoring for standardized PAP tests to the next, which could explain a few of the lower scores for those participants in their RCM tests. All of the participants, however, remained >−1.0 SD of the normative values for their age (Table 1B), which indicates that the healthy cognitive status of each participant was steady. Because on-site examinations in the Hillblom Network were suspended in accordance with public health restrictions during the COVID-19 pandemic, the availability of participants to recruit for RCM data collection was impacted. Although we aimed for an interval closer to 18 months, on average, between a participant’s PAP and RCM sessions, we believe the RCM scores across all eight tasks support our conclusion that the actual, longer interval was not a factor in the results for either task similarity or task comparability.

### The Role of Cognitive Screeners

The development and standardization of neuropsychological assessments over the past 50 years underlie the fundamental basis for how clinicians diagnose signs of cognitive decline in individual patients and how basic behavioral research organizes its broader insights about aging-related cognitive change. The manner of these neuropsychological assessments, which all have been collected in-person, has provided trained clinical staff with the opportunity to observe nuance highly valuable for the rich interpretation of each patient’s test data. For research purposes, validation of normative scores on standardized cognitive measures by age level opened the area of aging studies in cognitive neuroscience. The limitations of the in-person neuropsychological exam with PAP tests, however, arise from its limited access for many participants and its consequent lack of generalizability across the population. Because neuropsychological exams are administered as a lengthy battery of tests in sessions lasting three or more hours and conducted at specialized centers, both feasibility and practicality of the exams represent substantial obstacles for many older adults, particularly those living outside of major urban centers and/or those without access to insurance coverage for specialized healthcare. Indeed, the NIH Toolbox for Assessment of Neurological and Behavioral Function was implemented and then expanded through other studies as an effort to mitigate these limitations (Gershon et al., 2013; Flores et al., 2017), in particular the shortcoming that in-clinic neuropsychological batteries had been normed on homogenous non-diverse populations from a narrow range of ethnic, language, and cultural backgrounds. Another focus for improvement of neuropsychological assessment tools concerns the use of a standardized measure of reading comprehension (Fletcher and Grigorenko, 2017), which might reveal comorbidities that affect older adults’ verbal memory and fluency (Lebowitz et al., 2016).

Current digital technology may facilitate the development of platforms that can conduct, assess and score cognitive performance remote from the laboratory and do so reliably on a large scale (Koenig et al., 2019, 2021). The advent of secure and reliable remote data collection has already positively impacted therapeutic behavioral interventions (Gold et al., 2022), which will likely have a large focus on studies in cognitive aging (Ziegler et al., 2022). Regardless of the study scale, however, standardized assessments of older research participants’ cognitive performance will remain necessary for inclusion criteria for study enrollment and the stratification of individual data in the group analysis of behavioral results. Whether applied in large-scale remote studies or traditional in-lab studies, useful and comparable cognitive assessments can be collected remotely, as we show here with RCM.

Very important benefits from such a remote cognitive screener are vastly expanded reach for and inclusivity in recruitments to behavioral research. The validity of study designs and generalizability of conclusions from much of behavioral research, which have come under great scrutiny because of the shortcomings of smaller sample sizes and “inadequate rigor” (Kilkenny et al., 2009; Xu et al., 2022), can be improved with larger datasets demonstrated to be from a broader cross-section of the target population. But societal changes will be needed to accomplish improved outreach. Key factors are much broader realization of open-access software, availability of free internet, and efforts to increase ease of use for older adults (Martinho et al., 2022), and these factors will need to involve more than simply the commitment of behavioral researchers.

It is remote data collection functionality, which is a large step beyond mere digital functionality, that enables RCM or an equivalent application to improve outreach in the immediate community, take the most advantage of social media recruiting tools to target under-represented cohorts in research, and offer access to all users on the world-wide-web. The broadest useful application of a cognitive screener such as RCM turns in large part on its comparability and equivalence to assessments previously standardized in strata of population means with data collected using previous technology during bedside examinations. Considering the robust effects reported here for comparability of tests in long-term memory, language fluency, and set-shifting, we propose that RCM proves the concept for a remote digital screener as an effective alternative to in-person PAP tests for cognitive assessments in the healthy aging population.

## Data Availability Statement

The raw data supporting the conclusions of this article will be made available by the authors, without undue reservation.

## Ethics Statement

The studies involving human participants were reviewed and approved by the UCSF Committee on Human Research IRB #20-31812. The patients/participants provided their written informed consent to participate in this study.

## Author Contributions

PW designed the research, analyzed the data, and wrote the manuscript. JR designed the research, collected and analyzed the data, and wrote the manuscript. MA developed the digital procedure, collected and analyzed the data, and wrote the manuscript. RA-S developed the RCM digital program. AG provided guidance on the research and wrote the manuscript. All authors contributed to the article and approved the submitted version.

## Conflict of Interest

JR was employed by the Ochsner Health. The remaining authors declare that the research was conducted in the absence of any commercial or financial relationships that could be construed as a potential conflict of interest.

## Publisher’s Note

All claims expressed in this article are solely those of the authors and do not necessarily represent those of their affiliated organizations, or those of the publisher, the editors and the reviewers. Any product that may be evaluated in this article, or claim that may be made by its manufacturer, is not guaranteed or endorsed by the publisher.

## Footnotes

1
https://memory.ucsf.edu/research-trials/research/brain-aging-study
